# *miR-205* enhances radiation sensitivity of prostate cancer cells by impairing DNA damage repair through PKCε and ZEB1 inhibition

**DOI:** 10.1186/s13046-019-1060-z

**Published:** 2019-02-04

**Authors:** Rihan El Bezawy, Stella Tinelli, Monica Tortoreto, Valentina Doldi, Valentina Zuco, Marco Folini, Claudio Stucchi, Tiziana Rancati, Riccardo Valdagni, Paolo Gandellini, Nadia Zaffaroni

**Affiliations:** 1 Molecular Pharmacology Unit, Department of Applied Research and Technological Development, Fondazione IRCCS Istituto Nazionale dei Tumori di Milano, Via Amadeo 42, 20133 Milan, Italy; 2 Medical Physics Unit, Fondazione IRCCS Istituto Nazionale dei Tumori di Milano, 20133 Milan, Italy; 3 Prostate Cancer Program, Fondazione IRCCS Istituto Nazionale dei Tumori di Milano, 20133 Milan, Italy; 4 Radiation Oncology 1 Unit, Fondazione IRCCS Istituto Nazionale dei Tumori di Milano, 20133 Milan, Italy

**Keywords:** Prostate Cancer, miR-205, Radiosensitivity, PKCε

## Abstract

**Background:**

Radiotherapy is one of the main treatment options for non-metastatic prostate cancer (PCa). Although treatment technical optimization has greatly improved local tumor control, a considerable fraction of patients still experience relapse due to the development of resistance. Radioresistance is a complex and still poorly understood phenomenon involving the deregulation of a variety of signaling pathways as a consequence of several genetic and epigenetic abnormalities. In this context, cumulative evidence supports a functional role of microRNAs in affecting radioresistance, suggesting the modulation of their expression as a novel radiosensitizing approach. Here, we investigated for the first time the ability of miR-205 to enhance the radiation response of PCa models.

**Methods:**

miR-205 reconstitution by a miRNA mimic in PCa cell lines (DU145 and PC-3) was used to elucidate miR-205 biological role. Radiation response in miRNA-reconstituted and control cells was assessed by clonogenic assay, immunofluorescence-based detection of nuclear γ-H2AX foci and comet assay. RNAi was used to silence the miRNA targets PKCε or ZEB1. In addition, target-protection experiments were carried out using a custom oligonucleotide designed to physically disrupt the pairing between the miR-205 and PKCε. For in vivo experiments, xenografts generated in SCID mice by implanting DU145 cells stably expressing miR-205 were exposed to 5-Gy single dose irradiation using an image-guided animal micro-irradiator.

**Results:**

miR-205 reconstitution was able to significantly enhance the radiation response of prostate cancer cell lines and xenografts through the impairment of radiation-induced DNA damage repair, as a consequence of PKCε and ZEB1 inhibition. Indeed, phenocopy experiments based on knock-down of either PKCε or ZEB1 reproduced miR-205 radiosensitizing effect, hence confirming a functional role of both targets in the process. At the molecular level, miR-205-induced suppression of PKCε counteracted radioresistance through the impairment of EGFR nuclear translocation and the consequent DNA-PK activation. Consistently, disruption of miR-205-PKCε 3’UTR pairing almost completely abrogated the radiosensitizing effect.

**Conclusions:**

Our results uncovered the molecular and cellular mechanisms underlying the radiosensitizing effect of miR-205. These findings support the clinical interest in developing a novel therapeutic approach based on miR-205 reconstitution to increase PCa response to radiotherapy.

## Background

Radiotherapy is one of the main treatment options for non-metastatic prostate cancer (PCa) [1]. Although treatment technical optimization has greatly improved local tumor control, a considerable fraction of patients still experience relapse due to development of resistance [2]. Radioresistance is a complex and still poorly understood phenomenon involving the deregulation of a variety of signaling pathways as a consequence of several genetic and epigenetic abnormalities [3]. In this context, mounting evidence supports the ability of microRNAs (miRNAs) to interfere with different radioresistance-associated pathways, such as DNA-repair [4], epithelial-to-mesenchymal transition [5–8], and stemness [9]. miRNAs are endogenous small non-coding RNA molecules that negatively regulate gene expression [10]. The notion that miRNAs are heavily deregulated in PCa, together with the ability of a single miRNA to act as negative regulator of several genes and consequently modulate multiple cellular processes, has lead to increasing interest in defining a functional association between miRNA expression and radiation response [11].

Thus far, most studies assessing the involvement of specific miRNAs in radiosensitivity/radioresistance profile of PCa were limited to in vitro cell lines and generated controversial results [11]. A few in vivo studies on human PCa xenografts identified miRNAs promoting radioresistance, such as *miR-620*, which regulates prostaglandin E2 levels through direct targeting of hydroxyprostaglandin dehydrogenase 15 [12], and *miR-95,* which targets the sphingolipid phosphatase SGPP1 [13]. In the other hand, *miR-145* and *miR-890* were shown to increase radiation sensitivity of human PCa xenografts through down-regulation of multiple DNA repair genes [14, 15]. More recently, we demonstrated that *miR-875-5p* significantly enhances the radiation response of both in vitro and in vivo PCa experimental models by concomitantly counteracting epithelial-to-mesenchymal transition (EMT) and impairing DNA damage repair through the suppression of the EGFR-ZEB1 axis [16].

Here, we investigated the ability of *miR-205* to radiosensitize human PCa preclinical models. A lower *miR-205* expression was consistently found in PCa compared with matched normal prostate tissues in different studies [17–19]. In addition, we previously demonstrated that *miR-205* is essential for maintenance of the basal membrane in prostate epithelium [20], and that it blocks tumor-driven activation of surrounding fibroblasts by reducing secretion of the pro-inflammatory cytokine IL-6 [21], overall supporting a miRNA oncosuppressive function in PCa. The possible relevance of *miR-205* for PCa radiation response is based on our previous observation that its reconstitution in PCa cells counteracts EMT [17] and increases the antitumor activity of the DNA damaging agent cisplatin in vitro and in vivo, as a consequence of autophagy impairment [22], as well as on the reported evidence that PKCε, a direct *miR-205* target [17], plays a role in the nuclear translocation of EGFR, which is lost upon PKCε knockdown thus impairing DNA-double strand break (DSB) repair [23]. Consistently, results from this study indicate that *miR-205* reconstitution increases the radiation response of human PCa in vitro and in vivo models through the repression of the PKCε-EGFR-DNA-PK axis.

## Materials and methods

### Experimental models

The human DU145 and PC-3 PCa cell lines were purchased from the American Type Culture Collection (ATCC, Manassas, VA, USA) and maintained in RPMI-1640 medium supplemented with 10% fetal bovine serum (FBS). Cell lines were authenticated and periodically monitored by genetic profiling using short tandem repeat analysis (AmpFISTR Identifiler PCR amplification kit, Thermo Fisher Scientific Inc., Waltham, MA, USA).

### Cell transfection

Cells seeded at the appropriate density were transfected for 4 h with 20 nM mirVana miRNA mimic and negative control molecules (Thermo Fisher Scientific Inc) or with 20 nM siRNA molecules using Lipofectamine 2000 (Thermo Fisher Scientific Inc), according to the manufacturer’s instructions. In miR-Mask experiments, 20 nM PKCε-miScript Target Protector (Qiagen, Hilden, Germany) was transfected alone or in combination with *miR-205* mimic. SiRNAs targeting PKCε, ZEB1, LAMP3 and RAB27A were designed using siMAX Design Software and synthesized by Eurofin MWG Operon (Ebersberg, Germany). A control siRNA with no homology to any known human mRNA was also used. Hereafter, *miR-205* synthetic mimic will be referred to as miR-205, negative mock control oligomer as Neg, PKCε-miScript Target Protector as miR-Mask, PKCε siRNA as siPKCε, ZEB1 siRNA as siZEB1, LAMP3 siRNA as siLAMP3, RAB27A siRNA as siRAB27A and control siRNA as siCTRL. DU145 clones stably expressing *miR-205* were previously established as described in [22] and will be referred to as Vec miR-205 and cell stably transfected with negative control as Vec Neg.

### Clonogenic assay

Transfected cells were exposed to increasing doses (2–8 Gy) of irradiation delivered as a single dose using the 137Cs γ-irradiator IBL-437 (dose rate 5.2 Gy/min). Cells were then seeded at increasing density (500–8000 cells/well), in triplicate, in 6-well plates in RPMI medium containing 10% FBS. After 10 and 14 days, DU145 and PC-3 colonies, respectively, were fixed with 70% ethanol, stained with crystal violet in 70% ethanol, and counted. The plating efficiency was calculated as the ratio of the number of colonies (consisting of at least 50 cells) to the number of seeded cells. The surviving fraction was calculated as the ratio of the plating efficiency of the irradiated cells to that of the non-irradiated ones. Triplicate wells were set up for each condition.

### In vivo experiments

All animal experiments were approved by the Ethics Committee for Animal Experimentation of Fondazione IRCCS Istituto Nazionale dei Tumori. Ten million DU145 cells (Vec Neg and Vec miR-205 clones) were injected into the right flank of eight-week-old male SCID mice, and when tumors reached ~ 100 mm^3^ (Width^2^ x Length/2), mice were randomly assigned to control or radiation treatment groups (*n* = 8). Mice received 5 Gy single dose irradiation using a micro-CT/microirradiator (225Cx, Precision X-ray).

### miRNA and gene expression analysis

Quantification of *miR-205* and mRNA expression levels was assessed by qRT-PCR using the following TaqMan microRNA or gene expression assays (Thermo Fisher Scientific Inc): *miR-205,*000,509; PKCε Hs00942886_m1 and ZEB1 Hs00232783_m1. Amplifications were run on the 7900HT Fast Real-Time PCR System. Data were analyzed by SDS 2.2.2 software (Thermo Fisher Scientific Inc) and reported as relative quantity with respect to a calibrator sample using the 2-∆∆Ct method. RNU48 (PN4427975) and GAPDH (PN4326317E) were used as endogenous controls.

### Immunoblotting analyses

For immunoblotting, 20 μg of cell lysates was fractioned by SDS-PAGE and transferred onto nitrocellulose membranes using standard protocols. Equal protein loading was verified by Ponceau staining. Filters were blocked in PBS-Tween-20/0.5% skim milk and probed overnight with specific antibodies for PKCε (ab63638 and ab124806, Abcam, Cambridge, United Kingdom), EGFR (sc-03, Santa Cruz), pEGFR-T654 (ab78283, Abcam), ZEB1 (sc-10,572, Santa Cruz), DNA-PK (MS 423-PO, Thermo Fisher Scientific Inc), DNA-PK-T2609 (ab174576, Abcam). β-actin (a2066, Sigma-Aldrich, St. Louis, MO) or Vinculin (V9131, Sigma-Aldrich) and HDAC (877–616-cell, Cell Signaling) were used as equal protein loading controls. For phospho-DNA-PK evaluation, 48 h after transfection, cells were harvested at 30 and 60 min for subcellular protein fractionation by Subcellular Protein Fractionation Kit (Thermo Fisher Scientific). The filters were then incubated with the secondary peroxidase linked whole antibodies. Bound antibody was detected using the Novex ECL, HRP Chemiluminescent substrate Reagent Kit (Thermo Fisher Scientific Inc). For the preparation of figures, we cropped the original western blots to generate the figure panels with the relevant lanes. Cropped images were then subjected to uniform image enhancement of contrast and brightness. Molecular weights were determined using the colorimetric Precision Plus Protein Standard (Bio-Rad) and standard protein bands were removed from the chemiluminescent blot image.

### Immunofluorescence

Cells grown on glass coverslips were fixed with 4% formaldehyde and permeabilized with cold methanol/acetone solution. Cells were probed with FITC-labeled phalloidin (P5282; Sigma- Aldrich) or with primary antibody for phospho-Histone H2AX (ab11174, Abcam) and subsequently with Alexa Fluor488-labeled or Alexa Fluor594-labeled (Thermo Fisher Scientific Inc) secondary antibodies for 1 h at room temperature. Nuclei were counterstained with DAPI (Thermo Fisher Scientific Inc). Images were acquired by Nikon Eclipse E600 microscope using ACT-1 software (Nikon) and processed with ImageJ.

### Comet assay

The alkaline Comet assay (Trevigen Inc., Bologna, Italy) was performed on transfected cells 4 h from irradiation (4 Gy), according to the manufacturer’s instructions. Cells were suspended in low melting agarose and layered on microscope slides. Cells were then lysed to release the DNA. Electrophoresis was carried out under alkaline conditions. After electrophoresis for 10 min at 1 V/cm, slides were washed in water and dehydrated with ethanol, air-dried and then DNA was stained with SYBR Green. Comets were imaged using a fluorescence microscope equipped with a video camera (Jai Pulnix, Sunnyvale, CA), and quantitative assessment of DNA damage was obtained using the Comet Assay IV software (Perceptive Instruments, Suffolk, UK). Tail moments were determined by counting at least 200 comets/condition.

### Statistical analyses

Data are shown as mean values ± SD from at least three independent experiments. Statistical analysis was performed by two-tailed Student’s *t* test. *P-*values < 0.05 were considered statistically significant.

## Results

### *miR-205* enhances PCa cell sensitivity to radiation

**Fig. 1:**
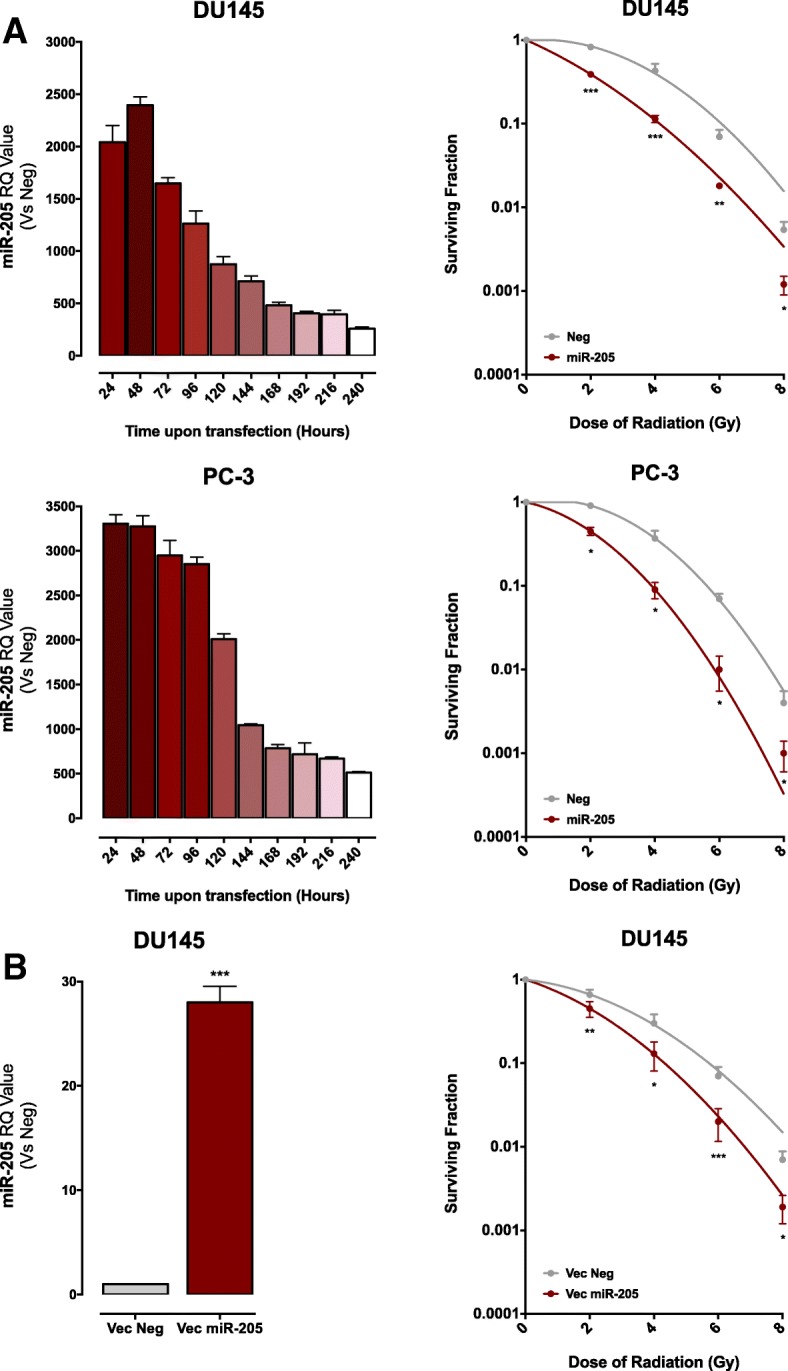
*miR-205* enhances PCa cell sensitivity to radiation. (**a**) (*Left panel*) qRT-PCR showing *miR-205* expression kinetics normalized to *RNU-48* in DU145 and PC-3 cells up to 240 h upon *miR-205* reconstitution, compared to control cells. Data are reported as relative quantity (RQ) ± SD with respect to Neg cells. All RQ values are statistically significant considering *p* < 0.0001. (*Right panel*) Clonogenic cell survival of DU145 and PC-3 cells transiently transfected with *miR-205* or Neg and exposed to increasing doses of irradiation (2, 4, 6 and 8 Gy). The surviving fraction is reported as mean ± SD values from 3 independent experiments. (**b**) (*Left panel*) qRT-PCR showing *miR-205* amount normalized to *RNU-48* in Vec *miR-205*-transfected DU145 cells, compared to control cells. Data are reported as relative quantity (RQ) ± SD with respect to Neg cells. (*Right panel*) Clonogenic cell survival of DU145 cells stably transfected with *miR-205* or Neg vector and exposed to irradiation (2, 4, 6 and 8 Gy). The surviving fraction is reported as mean ± SD values from 3 independent experiments. The level of significance was represented as *p < 0.05, **p < 0.01, ***p < 0.001, Student’s t-test

To assess the potential of *miR-205* as a modulator of radiation sensitivity of PCa cells, we adopted a gain-of-function approach. Transfection of DU145 and PC-3 cell lines – which inherently express almost undetectable levels of the miRNA [17] – with a *miR-205* synthetic mimic resulted in its persistent endogenous expression, still appreciable after ten days, being the values of relative expression highly significant, albeit reduced over time. (Fig. 1A, *left panels*). Consistent with previous data [17], restoring the expression of *miR-205* did not appreciably affect cell proliferative potential, as indicated by the plating efficiencies of transfected DU145 and PC-3 cells (0.20 and 0.29, respectively), which were superimposable to those of corresponding negative-control transfectants (0.22 and 0.28, respectively). Interestingly, *miR-205* reconstitution resulted in an increased sensitivity of both cell lines to radiation, as suggested by the reduction in their clonogenic cell survival compared to controls, which was statistically significant along the whole range of doses (Fig. 1a, *right panels*).

Notably, the radiosensitizing effect was maintained in a polyclonal population of DU-145 cells stably transfected with a vector containing the hairpin precursor sequence of *miR-205* [22], where the persistent miRNA overexpression, although to a lower extent compared to transiently reconstituted DU145 cells (Fig. 1b, *left panel*), was sufficient to induce a significant enhancement of radiation response (Fig. 1b, *right panel*). Again, *miR-205* stable reconstitution did not affect the clonogenic potential of DU145 cells (plating efficiencies, 0.13 and 0.15 in miRNA-205- and negative control-transfected cells, respectively).

These results suggest that *miR-205* is endowed with a radiosensitizing potential in PCa cell models.

### *miR-205* impairs cell repair of radiation-induced DNA damage

**Fig. 2:**
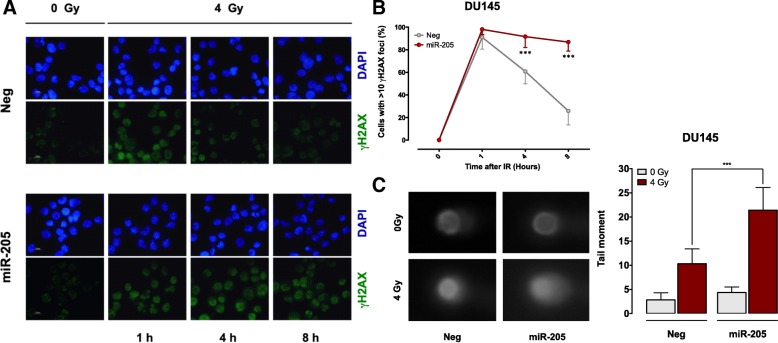
*miR-205* impairs cell repair of radiation-induced DNA damage. (**a**) Representative immunofluorescence images of nuclear γH2AX foci (cell nuclei: blue; γH2AX foci: green) in DU145 cells at 1, 4 and 8 h after exposure to 4 Gy of irradiation. (**b**) Kinetics of γH2AX foci resolution in Neg- or *miR-205*-reconstituted DU145 cells, expressed as mean number of cells containing > 10 γH2AX foci at 1, 4 and 8 h after exposure to 4 Gy of irradiation (IR). Data are reported as mean ± SD values from 3 independent experiments. (**c**) *(Left panel)* Photomicrographs showing formation of Comets in Neg- or *miR-205*-transfected DU145 cells at 4 h after exposure to 4 Gy of irradiation. *(Right panel)* Quantification of tail moments counted in at least 200 cells per group, reported as mean ± SD values from 3 independent experiments. The level of significance was represented as ***p < 0.001, Student’s t-test

To test whether the observed radiosensitizing effect was based on *miR-205* ability to interfere with cell mechanisms of DNA repair, we assessed the persistence of radiation-induced damage by evaluating the kinetics of accumulation and removal of γH2AX foci, a specific marker of the presence of DNA-DSBs [24]. Immunofluorescence staining of γH2AX showed that the treatment induced an extensive and comparable DNA damage in both *miR-205*-reconstituted and control cells, as indicated by the presence of high positivity (> 10 foci/cell) to γH2AX in > 90% of cells at 1 h from radiation treatment (Fig. 2a and b). However, γH2AX foci resolution at 4 and 8 h after irradiation was markedly delayed in *miR-205*-reconstituted cells, indicating that the miRNA impairs cell proficiency in recovering from radiation-induced DNA-DSBs (Fig. 2a and b). Consistently, when assessing DNA damage at a single cell level by comet assay, *miR-205*-reconstituted cells presented significantly extended comet tail moments, as detected at 4 h after irradiation, reflecting the presence of a markedly higher amount of unrepaired DNA breaks with respect to control cells (Fig. 2c).

These findings support the hypothesis that *miR-205* impairs the ability of PCa cells to repair radiation-induced DNA damage.

### *miR-205* enhances in vivo response to radiotherapy in PCa xenografts

**Fig. 3:**
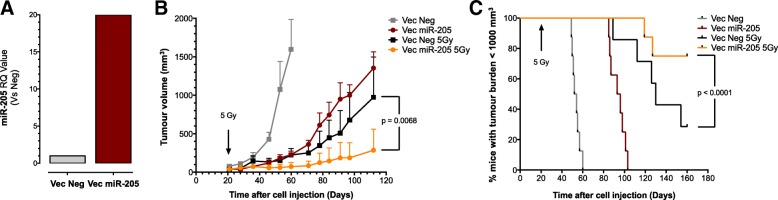
*miR-205* enhances PCa xenograft response to radiation therapy**.** DU145 cells stably transfected with *miR-205* or Neg vector (1 × 10^7^) were implanted subcutaneously into the right flank of SCID mice. When tumors reached ~ 100 mm^3^, mice were randomly assigned to four groups (8 mice/group) and treated with 5 Gy single dose irradiation locally delivered to the tumor. (**a**) qRT-PCR showing *miR-205* amount normalized to *RNU-48* in Vec *miR-205*-tumours, compared to control ones. (**b**) Tumor growth volume (mm^3^) measured with a Vernier caliper on indicated days after cell injection. (**c**) Kaplan-Meier plot of mouse tumor growth to 1000 mm^3^

Our in vitro findings were challenged in the in vivo setting by subcutaneously transplanting DU145 cells stably transfected with *miR-205*-expressing vector and control vector into SCID mice to generate xenografts. The enhanced expression of the miRNA was confirmed by qRT-PCR (Fig. 3a). Mice were then exposed to 5 Gy single dose irradiation at 20 days after cell inoculum.

No differences were appreciable in the tumor take rate of *miR-205* and control xenografts (which was 100% in all experimental groups), although the growth of non-irradiated xenograft tumors originated from *miR-205*-reconstituted DU145 cells was delayed compared to those arising from control cells (Fig. 3b and c). Since a comparable proliferation rate (in terms of plating efficiency in the clonogenic assay) was observed in vitro for both cell lines, such a growth delay was likely due to limited local invasive capabilities of *miR-205* expressing cells, consistent with our previous finding indicating the ability of the miRNA to counteract EMT and impair migration and invasive properties in DU145 cells following transfection with a *miR-205* synthetic precursor [17].

Interestingly, *miR-205* enhanced the effect of radiation also in vivo, as indicated by a statistically significant reduction in tumour growth upon irradiation compared to controls (Fig. 3b) and, especially, by the significantly increased time for DU145 xenografts to reach 1000 mm^3^ tumour burden with respect to controls (Fig. 3c).

### Repression of the PKCε-EGFR-DNA-PK axis as main determinant of *miR-205*-mediated radiosensitization

To dissect the molecular determinants underlying *miR-205*-induced radiosensitizing effect in PCa cells, we explored the possible role of miRNA target genes relevant to radiation response. Target choice was based on previous findings indicating that PKCε plays a central role in radiation response of A549 lung carcinoma cells by inducing nuclear translocation of EGFR and activation of DNA-PK [23], together with the evidence that *miR-205* increased radiation response of breast cancer models through ZEB1 suppression and consequent inhibition of homologous recombination (HR) repair of DNA-DSBs [4].

**Fig. 4:**
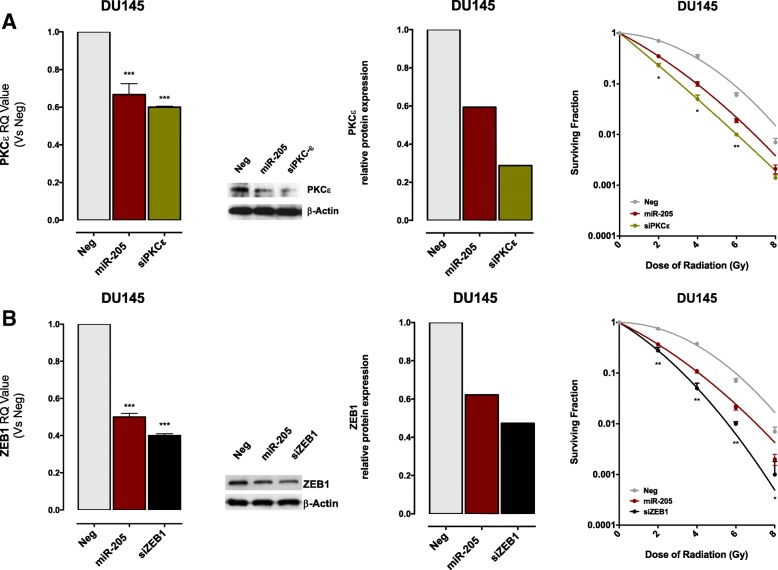
*PKCε* and *ZEB1* down-regulation phenocopies *miR-205*-induced radiosensitization. (**a**) (*Left panel*) qRT-PCR showing *PKC****ε*** mRNA amount in *miR-205-* or siPKCε-transfected DU145 cells, compared to control cells, normalized to *GAPDH*. Data are reported as relative quantity (RQ) ± SD with respect to Neg cells. (*Middle panel*) Western blot analysis showing PKCε protein amount in DU145 cells upon transfection with Neg, *miR-205* or siPKCε. β-Actin was used as equal protein loading controls. Cropped images of selected proteins are shown. (*Right panel*) Clonogenic cell survival of DU145 cells transfected with *miR-205* or siPKCε. The surviving fractions following the indicated doses of irradiation are reported as mean ± SD values from 3 independent experiments. (**b**) (*Left panel*) qRT-PCR showing *ZEB1* mRNA amount in *miR-205-* or siZEB*-*transfected DU145 cells, compared to control cells, normalized to *GAPDH*. Data are reported as relative quantity (RQ) ± SD with respect to Neg cells. (*Middle panel*) Western blot analysis, and corresponding relative quantification, showing ZEB1 protein amount in DU145 cells upon transfection with Neg, *miR-205* or siZEB1. β-Actin was used as equal protein loading controls. Cropped images of selected proteins are shown. (*Right panel*) Clonogenic cell survival of DU145 cells transfected with *miR-205* or siZEB1. The surviving fractions are reported as mean ± SD values from 3 independent experiments. The level of significance was represented as *p < 0.05, **p < 0.01, ***p < 0.001, Student’s t-test

Phenocopy experiments were carried out to assess the radiation response of DU145 cells upon knockdown of PKCε or ZEB1 with specific siRNAs able to reduce both mRNA and protein at levels comparable to those observed following *miR-205* reconstitution (Fig. 4a and b, *left panels*). Down-regulation of either gene was able to induce a radiosensitizing effect comparable (or slightly greater, although not significantly different) to that observed following *miR-205* reconstitution, as indicated by the clonogenic cell survival curves following exposure to increasing radiation doses (Fig. 4a and b, *right panels*).

While *miR-205*-induced inhibition of ZEB1 and of the ubiquitin-conjugating enzyme Ubc13, and the consequent decrease in the ability to repair DNA damage by the homologous recombination (HR) pathway, has been exhaustively documented as a main responsible for the miRNA radiosensitizing effect in breast cancer cells [4], the role of PKCε in *miR-205*-mediated increased radiation response of tumour cells has never been investigated.

**Fig. 5:**
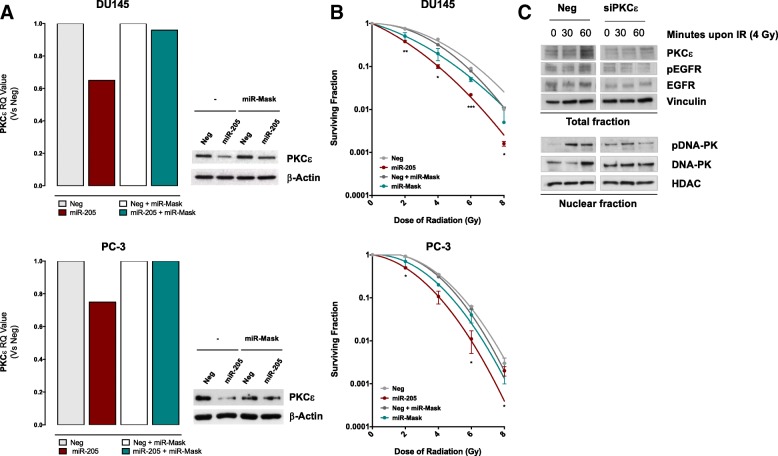
Repression of the PKCε-EGFR-DNA-PK axis as main determinant of *miR-205*-mediated radiosensitization. (**a**) (*Left panel*) qRT-PCR showing PKCε mRNA amount in DU145 or PC-3 cells transfected with *miR-205,* in the presence or absence of miR-Mask*,* compared to control cells, normalized to *GAPDH*. Data are reported as relative quantity (RQ) ± SD with respect to Neg cells. (*Right panel*) Western blot analysis showing PKCε protein amount in DU145 and PC-3 cells upon *miR-205*-reconstitution in the presence or absence of miR-Mask. β-Actin was used as equal protein loading controls. Cropped images of selected proteins are shown. (**b**) Clonogenic cell survival of DU145 (*upper panel*) or PC-3 (*lower panel*) cells transfected with *miR-205*, miR-Mask or both. The surviving fractions are reported as mean ± SD values from 3 independent experiments. (**c**) Forty-eight h after transfection with Neg or siPKCε, DU145 cells were exposed to 4 Gy and 30 and 60 min later harvested for total protein collection or subcellular protein fractionation and western blot analysis. Results showing the total amount of PKCε and phospho-EGFR (*upper panel*) and of nuclear phospho-DNA-PK levels (*lower panel*) are reported. Vinculin and HDAC were used as control for total and nuclear fractions, respectively. Cropped images of selected proteins are shown. The level of significance was represented as *p < 0.05, **p < 0.01, ***p < 0.001, Student’s t-test

Our finding that PKCε knockdown affects PCa cell radiosensitivity similarly to *miR-205* restoration does not per se demonstrate that miRNA-induced radiosensitization directly relies on PKCε down-regulation. To address this point, a target protection approach was pursued. Specifically, DU145 and PC-3 cells were co-transfected with *miR-205* mimic and a miR-Mask, a custom oligonucleotide designed to be fully complementary to *miR-205* binding site within PKCε 3’UTR, to assess whether the disruption of miRNA-target interaction could abolish *miR-205* radiosensitizing effect. Notably, the miR-Mask was able to almost completely restore PKCε transcript and protein expression levels, thus confirming PKCε as a direct target of *miR-205* (Fig. 5a). Interestingly, the presence of miR-Mask abrogated, although partially, *miR-205* radiosensitizing effect in both PCa cell lines, substantiating a scenario proposing PKCε down-regulation as an important determinant of *miR-205*-induced enhancement of radiation response (Fig. 5b).

PKCε has been reported to be up-regulated upon radiation exposure in A549 lung carcinoma cells [25] and to stimulate radiation-induced damage repair through EGFR phosphorylation and nuclear accumulation, which in turn phosphorylates and activates DNA-PK thus triggering non-homologous end joining (NHEJ) pathway [23]. Consistently, we found that at 30 and 60 min following exposure to 4 Gy irradiation, DU145 control cells displayed enhanced levels of PKCε, pEGFR (T654) and nuclear pDNA-PK (T2609), which were not appreciable in *miR-205*-reconstituted cells (Fig. 5c).

**Fig. 6:**
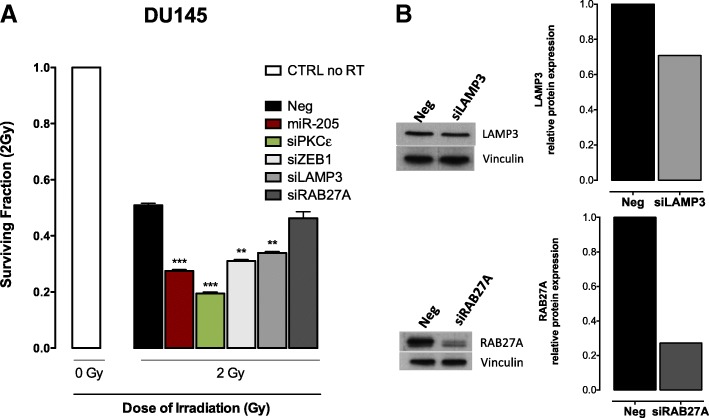
LAMP3 downregulation recapitulates miR-205 induced radiosensitizing effect. (**a**) Surviving fractions at 2 Gy of DU145 cells transfected with either *miR-205*, siPKCε, siZEB1, siLAMP3 or siRAB27A are reported as mean ± SD values from 3 independent experiments. The level of significance was represented as **p < 0.01, ***p < 0.001, Student’s t-test. (**b**) Western blot analysis, and corresponding relative quantification, showing LAMP3 (*upper panel*) and RAB27 (*lower panel*) protein amount in DU145 cells upon transfection with siLAMP3 or siZEB1, respectively. Vinculin was used as equal protein loading controls. Cropped images of selected proteins are shown

We have previously reported that miR-205 replacement in PCa cells induces an enhancement of cisplatin cytotoxic activity, as a consequence of autophagy impairment mediated by the downregulation of the lysosome-associated proteins LAMP3 and RAB27A [22]. Here, we found that the silencing of LAMP3, but not RAB27A, significantly enhanced the radiation response of DU145 cells, as indicated by the reduction of survival fractions at 2 Gy (Fig. 6a), independently of the level of protein expression inhibition (Fig. 6b).

Overall, our findings suggest that *miR-205* exerts its radiosensitizing effect mainly by impairing DNA repair pathways through the inhibition of ZEB1 and the suppression of PKCε-EGFR-DNA-PK axis, respectively, ultimately resulting in a decrease removal of radiation-induced DNA lesions and consequent enhancement of PCa cell susceptibility to radiation. Moreover, miR-205-induced impairment of the autophagy flux, mainly through LAMP3 downregulation, could represent an additional mechanism by which the miRNA exerts its radiosensitizing effect.

## Discussion

Results obtained in experimental models have demonstrated a direct involvement of selected miRNAs in controlling tumor radiation response, thus revealing an entirely new mechanism of radioresistance but also envisaging a possible novel approach of radiosensitization based on the modulation of specific miRNAs [26].

In this study, we showed that reconstitution of *miR-205*, the expression of which is down-regulated in primary and, even more, in metastatic PCa lesions [17–19], was able to enhance radiation response in both in vitro and in vivo PCa models. *miR-205* is known to exert oncosuppressive functions in PCa [17, 20–22], some of which are relevant to radiation-response. Specifically, *miR-205* was found to counteract EMT in PCa cells through the suppression of PKCε [17]. EMT was reported to be related to radioresistance in many cancers [5–8]. In this context, we and others already showed the relevance of some EMT-related miRNAs, including *miR-200c* [27], *miR-203* [28], *miR-204* [29] and *miR-875-5p* [16] in determining the radiation response of experimental tumor models.

Since it is well known that radiosensitivity depends in part on tumor cell kinetics, being G2-M the most radiosensitive phase of the cell cycle [30], the recently established role for PKCε in the control of mitotic spindle organization in transformed cell models [31] could also contribute to the *miR-205*-mediated radiosensitizing effect. Most importantly, PKCε also plays a role in the nuclear translocation of EGFR, a main mechanism of tumor radioresistance [23]. Specifically, it has been shown that radiation-induced nuclear EGFR presented PKCε-mediated increased phosphorylation at T654 [23]. EGFR contributes to radiation resistance, at least in part, by interacting with the catalytic subunit of DNA-PK and increasing the enzyme activity through stabilization of the phosphorylated forms of the protein at specific serine (S2056) and threonine (T2609) residues, which are essential for DNA double-strand break repair by NHEJ pathway [32]. Indeed, we found that siRNA-mediated PKCε down-regulation was able to phenocopy the radiosensitizing effect induced by *miR-205* and to reduce the accumulation of phosphoEGFR and phosphoDNA-PK. Consistently, the inhibition of *miR-205*-PKCε interaction through the use of a miRNA mask almost completely abolished the radiosensitizing effect of the miRNA as a consequence of the complete recovery of PKCε expression.

*miR-205* reconstitution in experimental models of human breast cancer, another tumor type characterized by a reduced expression of the miRNA [33], was found to improve the radiation response by directly targeting ZEB1 and the ubiquitin-conjugating enzyme Ubc13, thus inhibiting HR-mediated repair of DNA-DSBs [4]. Consistently, in this study we found a significant inhibition of ZEB1 expression, at both mRNA and protein level, following *miR-205* reconstitution in PCa cell lines. In addition, siRNA-mediated ZEB1 silencing was able to recapitulate *miR-205* radiosensitizing effect, thus confirming a functional role of ZEB1 in determining the radiation response also in PCa cells.

**Fig. 7:**
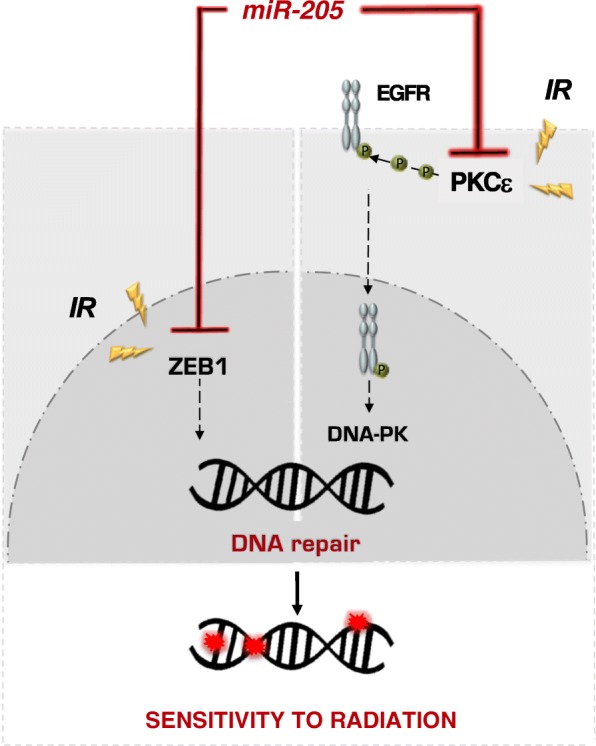
The working model of *miR-205* radiosensitizing effect. *MiR-205*-induced suppression of ZEB1 and PKCε leads to the impairment of DNA-repair, thus resulting in an enhancement of cell sensitivity to radiation

The emerging role of *miR-205* as a negative regulator of both NHEJ and HR repair pathways (Fig. 7) is supported by the significantly reduced clearance of radiation-induced DNA-DSBs we observed in miRNA-reconstituted PCa cells, in terms of increased residual γH2AX foci and extended comet tail moments at different interval following irradiation.

In addition, consistent with the previously reported ability of miR-205 to enhance the response of PCa to cisplatin treatment through autophagy impairment [22], results of this study suggest a possible role of LAMP3 downregulation in the miRNA-mediated enhancement of PCa cell radiation response.

Although data collected in PCa and breast cancer models support the clinical interest in developing a novel *miR-205*-based radiosensitization approach, the evidence that involvement of specific miRNAs in the onset of radiation resistance seems to be cell/tissue specific represents a constraint for the clinical exploitation of miRNA-based therapeutic molecules. In this context, it was found that Sp1-mediated transcriptional activation of *miR-205* promotes radioresistance in esophageal squamous cell carcinoma [34], where the miRNA was reported to exert oncogenic functions [35], and that *miR-205* determines the radioresistance of human nasopharyngeal carcinoma by directly targeting PTEN [36], thus envisaging a possible opposite role of the miRNA in controlling radiation response as a function of tumour cell type, based on the availability of specific targets.

## Conclusions

In conclusion, this is the first report indicating that miR-205 reconstitution enhances radiation response in prostate cancer cell and xenograft models. We also proposed that such an effect may mainly rely on DNA repair impairment as a consequence of PKCε and ZEB1 targeting, as suggested by the evidence that RNAi-mediated silencing of either gene was able to phenocopy miR-205 radiosensitizing effect. Cumulatively, our results support the clinical interest in developing a novel therapeutic approach based on miR-205 reconstitution to increase prostate cancer response to radiotherapy.

## Abbreviations

EGFR: Epidermal growth factor receptor
EMT: Epithelial-to-mesenchymal transition
HR: Homologous recombination
miRNA: microRNA
NHEJ: Non homologous end joining
PCa: Prostate cancer
PKCε: Protein kinase C epsilon
qRT-PCR: Quantitative real-time polymerase chain reaction
SCID: Severe combined immunodeficiency
siRNA: Small interfering RNA
ZEB1: Zinc finger E-box binding homeobox 1
